# Current News

**Published:** 1905-04

**Authors:** 


### Current News.

### IOWA STATE DENTAL SOCIETY.

THE forty-third annual meeting of the Iowa State Dental Society will be held in Des Moines, May 2, 3, and 4, 1905.

A programme of clinics and papers, promising more than usual interest, will be given. A cordial invitation is extended to the dental profession to attend.

C. W. BRUNER,
*Secretary.*

### CALIFORNIA STATE DENTAL ASSOCIATION.

AT a meeting of the board of trustees of the California State Dental Association, held in San Francisco, February 10, it was unanimously decided to adjourn the State meeting for 1905 in favor of the Lewis and Clarke Dental Congress, to be held at Portland, July 17, 18, 19, and 20, 1905.

JOSEPH LORAN PEASE,
*Corresponding Secretary.*

### PENNSYLVANIA STATE BOARD OF DENTAL EXAMINERS.

THE Board of Dental Examiners of Pennsylvania will conduct examinations simultaneously in Philadelphia and Pittsburg, June 6 to 9, 1905.

Applicants for examination must address

DR. N. C. SCHAEFFER,
*Secretary Dental Council.*

HARRISBURG, PA.

